# The Role of Brain-Derived Neurotrophic Factor (BDNF) in Neural Development and Cognitive Behavior in Pigeons: Advances and Future Perspectives

**DOI:** 10.3390/cimb48040384

**Published:** 2026-04-08

**Authors:** Guanhui Liu, Luyao Li, Su Wang, Jiarong Sun, Yongyan Han, Yaxuan Gao, Dongmei Han

**Affiliations:** School of Life Sciences and Food Engineering, Hebei University of Engineering, Handan 056038, China; liuguanhui.1986@163.com (G.L.); 13932094619@163.com (L.L.); wangsu_1023@163.com (S.W.); jiarong8886@163.com (J.S.); m18547695360@163.com (Y.H.); goyx201@163.com (Y.G.)

**Keywords:** Brain-Derived Neurotrophic Factor (BDNF), synaptic plasticity, epigenetic regulation, non-coding RNAs, neural development, spatial navigation, homing behavior, pigeon

## Abstract

Brain-Derived Neurotrophic Factor (BDNF), a key member of the neurotrophin family, is critically involved in neuronal survival, synaptic plasticity, learning, and memory. While its roles in mammals have been extensively documented, the molecular regulatory mechanisms governing *BDNF* expression and its causal contributions to complex cognitive behaviors remain poorly understood in non-mammalian vertebrates—particularly for the domestic pigeon (*Columba livia domestica*), a species distinguished by its remarkable spatial navigation and homing capabilities. This review synthesizes the current evidence on BDNF in the pigeon central nervous system across five thematic domains: molecular structure and isoform diversity, transcriptional and epigenetic regulatory networks, involvement in neural development, associations with cognitive and navigational behaviors, and potential translational applications. A particular emphasis is placed on the region-specific and activity-dependent expression patterns of BDNF in brain structures such as the hippocampal formation (HF), optic tectum, and striatum, and their functional relevance to visual processing, homing behavior, and stress adaptation. To date, most findings remain correlational; therefore, establishing a mechanistic understanding necessitates the integration of advanced methodologies—including single-cell omics, CRISPR-based gene editing, and high-resolution behavioral phenotyping—to causally link BDNF dynamics, neural circuit modulation, and spatial cognition. This synthesis aims to bridge gaps in comparative neurobiology, inform molecular approaches to avian cognitive enhancement, and support evidence-based strategies for racing pigeon breeding and welfare assessment.

## 1. Introduction

Brain-Derived Neurotrophic Factor (BDNF) was first isolated in 1982 and has since been recognized as a key regulator of synaptic plasticity, neuronal survival, and activity-dependent gene expression in the mammalian central nervous system (CNS). In rodents and humans, BDNF signaling through tropomyosin receptor kinase B (TrkB) modulates long-term potentiation (LTP), dendritic arborization, and hippocampal-dependent memory formation, underpinning its role in learning, emotional processing, and neurodevelopmental stability [1,2].

Comparative studies have demonstrated that non-mammalian vertebrates also exhibit complex cognitive behaviors supported by conserved neurotrophic systems. The domestic pigeon displays sophisticated spatial navigation, visual discrimination, and temporal processing capabilities, making it a valuable model for investigating the neural basis of multisensory integration and homing behavior [3]. Despite the absence of a laminated neocortex, birds possess telencephalic structures with functional parallels to mammalian higher-order regions: the nidopallium caudolaterale (NCL) and the hippocampal formation (HF) are implicated in executive control and spatial memory, respectively, based on lesion, electrophysiological, and molecular evidence [4,5].

*BDNF* expression in the pigeon HF is upregulated following navigational training, and transcript levels correlate positively with task complexity and repetition frequency [3,5]. However, comprehensive characterization of the *BDNF* gene locus—including its exon–intron architecture, alternative promoter usage, and post-transcriptional regulation—remains incomplete in avian species. In contrast, these mechanisms have been extensively delineated in laboratory rodents, particularly the house mouse (*Mus musculus*) and the Norway rat (*Rattus norvegicus*), which serve as the primary mammalian models for dissecting BDNF-dependent neuroplasticity. Decades of research in these species have elucidated the roles of epigenetic modifications (e.g., DNA methylation at BDNF promoters), activity-regulated non-coding RNAs, and cell type-specific BDNF trafficking in sensory integration, synaptic plasticity, and memory consolidation [6,7,8]. In comparison, such molecular and cellular dimensions of BDNF regulation are poorly defined in birds.

This review summarizes the current knowledge on Brain-Derived Neurotrophic Factor (BDNF) in the pigeon nervous system, integrating findings across molecular, cellular, circuit, and behavioral levels to outline its involvement in neural development and cognitive processes. The discussion is organized into five thematic sections: the molecular features of BDNF, its transcriptional and post-transcriptional regulatory networks, its roles in neural development, its contributions to cognition and behavior, and current methodological limitations with directions for future investigation.

## 2. Molecular Foundations of BDNF

A comprehensive understanding of BDNF’s molecular biology in birds—particularly regarding gene regulation, protein processing, and isoform-specific signaling—remains limited compared to mammals. Due to the scarcity of direct experimental data in avian species, much of the current conceptual framework is inferred from well-established mechanisms in mammalian models. Below, we outline these conserved principles while explicitly highlighting knowledge gaps and the need for empirical validation in pigeons and other birds.

### 2.1. Gene Structure Characteristics

The *BDNF* gene exhibits a conserved genomic architecture across vertebrates, typically comprising multiple 5′ non-coding exons spliced to a single 3′ protein-coding exon [9,10]. In the domestic pigeon (*Columba livia domestica*), the *BDNF* locus is annotated on chromosome 5 (reference sequence NC_088606.1; ~42.9 kb), spanning from upstream regulatory regions to the transcriptional termination site. Current genome annotation predicts eight exons, of which seven are putative non-coding exons, each potentially associated with an alternative promoter, and a single downstream exon that harbors the complete open reading frame encoding the pre-proBDNF precursor protein [11]. This structural arrangement—though based on computational prediction—suggests a capacity for context-dependent transcriptional regulation through promoter choice and alternative splicing, as observed in mammals [9].

Compared to mammals, the pigeon *BDNF* locus is substantially more compact (~43 kb versus >70 kb in humans) [10,11], primarily due to shortened intronic regions—a feature consistent with the general genome compaction characteristics of avian lineages [12]. While the overall exon–intron scaffold appears conserved, cis-regulatory elements linked to the predicted non-coding exons likely exhibit species-specific sequence divergence. For example, the core promoter region corresponding to the activity-regulated exon IV in mammals shows evolutionary conservation [13]; however, adjacent enhancer or repressor motifs may have undergone lineage-specific modifications, potentially shaping stimulus-responsive transcriptional dynamics in birds. It should be emphasized that the functional equivalence of these predicted avian exons to their mammalian counterparts—including their promoter usage, splicing patterns, and regulatory roles—remains to be experimentally validated.

### 2.2. Protein Processing and Maturation of BDNF

**Figure 1:**
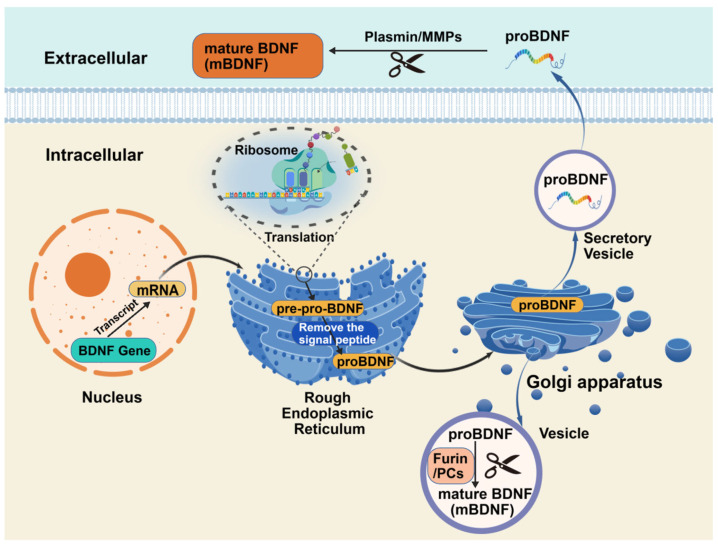
Mechanism of BDNF biosynthesis. Created with BioGDP (https://biogdp.com/). Created with BioGDP.com [27].

BDNF is synthesized as a precursor (pre-proBDNF) that undergoes sequential proteolytic cleavage to yield functionally distinct isoforms [14]. In mammals, nascent pre-proBDNF is translocated into the endoplasmic reticulum (ER), where signal peptide removal generates proBDNF. This precursor is trafficked through the Golgi and packaged into secretory vesicles [14,15]. Intracellular maturation to mature BDNF (mBDNF) is mediated by proprotein convertases (e.g., furin, PC1/3) in the trans-Golgi network or immature granules [16,17,18]. mBDNF is stored in dense-core vesicles and released via calcium-dependent exocytosis [14,16]. Alternatively, proBDNF may be secreted intact and cleaved extracellularly by serine proteases such as plasmin or matrix metalloproteinases (MMP-3/9) [16,19]. The balance between proBDNF and mBDNF critically determines signaling through the p75 neurotrophin receptor (p75NTR) (often pro-apoptotic) versus TrkB (pro-survival/plasticity) receptors (Figure 1) [20].

While this processing paradigm is well documented in rodents (e.g., Mus musculus, Rattus norvegicus) [21,22,23], its applicability to avian systems remains unverified. For example, reduced furin expression impairs mBDNF production and spatial memory in mice [21], and elevated MMP activity enhances synaptic potentiation via extracellular proBDNF conversion [22,23]. Whether similar enzymatic pathways operate in the pigeon brain—and whether they contribute to navigational plasticity—is currently unknown. Given the high expression of BDNF in the pigeon HF [24], elucidating the molecular machinery governing its isoform balance represents a key frontier for understanding avian cognitive neurobiology.

Complementing these molecular considerations, BDNF exhibits a region-specific expression pattern within the avian central nervous system, reflecting both evolutionarily conserved neurotrophic demands and lineage-specific functional specializations [25]. In the domestic pigeon, BDNF mRNA and protein are detectable across multiple forebrain and midbrain regions, including the telencephalon, diencephalon, and mesencephalon. Notably, higher transcript and protein levels are consistently reported in telencephalic structures implicated in spatial cognition and multimodal integration—such as the HF, medial striatum (MSt), and hyperpallium [24,26].

## 3. Expression Regulatory Networks of BDNF

### 3.1. Bidirectional Signaling Pathways

The functional versatility of BDNF is determined by both its regional expression pattern and the distinct biological activities of its proteolytic isoforms [2,28]. BDNF exists primarily as proBDNF and mBDNF, which engage different receptor systems and elicit opposing cellular responses [29].

mBDNF binds with a high affinity to the full-length tropomyosin receptor kinase B (TrkB-FL), a receptor tyrosine kinase [28,29]. Ligand binding induces TrkB dimerization and the autophosphorylation of intracellular tyrosine residues, leading to the recruitment and activation of downstream signaling effectors [28]. Three major pathways are predominantly activated: the mitogen-activated protein kinase/extracellular signal-regulated kinase (MAPK/ERK) cascade, the phosphatidylinositol 3-kinase/protein kinase B (PI3K/AKT) axis, and the phospholipase C-gamma (PLC-γ) pathway [28]. These signaling modules regulate neuronal survival, synaptic structure, and activity-dependent gene transcription [28,30]. The MAPK/ERK and PI3K/AKT pathways are associated with the synthesis, trafficking, and membrane insertion of synaptic proteins, processes that correlate with enhanced synaptic efficacy and cognitive performance. The PLC-γ pathway modulates intracellular calcium (Ca^2+^) flux [30], resulting in phosphorylation of the cAMP response element-binding protein (CREB), which in turn influences transcriptional programs involved in synaptic transmission.

**Figure 2:**
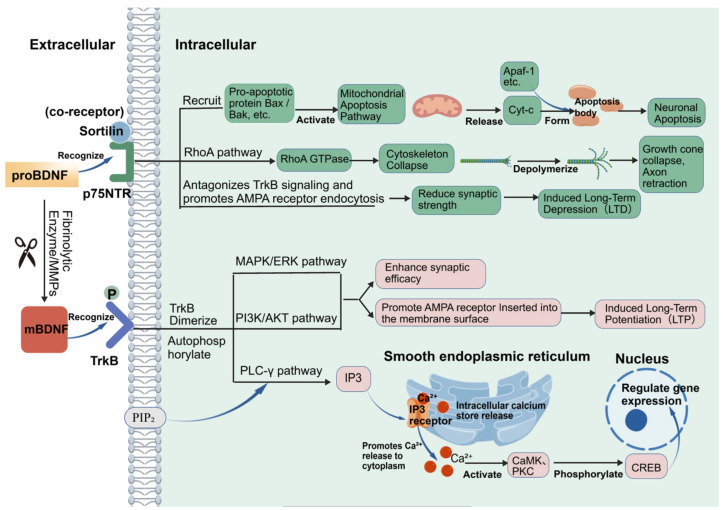
BDNF signaling pathway.Arrows indicate signaling flow. Green boxes highlight the p75NTR-mediated pathway (apoptosis/LTD) triggered by proBDNF, while pink boxes highlight the TrkB-mediated pathway (survival/LTP) activated by mBDNF. Created with BioGDP (https://biogdp.com/). Created with BioGDP.com [27].

In contrast, proBDNF preferentially interacts with the p75NTR [31]. This interaction activates intracellular cascades that promote neuronal apoptosis, axonal retraction, and long-term depression (LTD), thereby reducing synaptic strength and contributing to the negative regulation of neural connectivity [32]. The balance between mBDNF/TrkB-FL and proBDNF/p75NTR signaling thus shapes bidirectional outcomes in synaptic plasticity and neuronal viability (Figure 2).

The regulatory complexity of BDNF signaling is further shaped by an alternative splicing of the *NTRK2* gene, which encodes tropomyosin receptor kinase B (TrkB). In addition to the full-length, catalytically active isoform (TrkB-FL), truncated variants—predominantly TrkB-T1 and TrkB-T2—are widely expressed across the vertebrate nervous system [33,34,35]. These isoforms lack the intracellular tyrosine kinase domain and therefore cannot transduce canonical neurotrophic signals upon BDNF binding. Instead, they may act as dominant-negative modulators by sequestering extracellular BDNF or forming non-signaling heterodimers with TrkB-FL, thereby dampening downstream pathway activation [34,35].

Evidence from avian models supports the functional relevance of this isoform diversity: in the developing visual system, the spatially and temporally regulated expression of TrkB splice variants has been implicated in activity-dependent synaptic refinement [33]. However, despite the growing recognition of BDNF’s role in avian spatial cognition, the expression profile and functional contribution of TrkB isoforms in the pigeon HF—and specifically within navigation-related circuits—remain poorly defined. Insights from mammalian systems indicate that TrkB isoform balance can critically influence experience-dependent plasticity [36]. By analogy, the differential expression of TrkB isoforms in pigeons may constitute a previously underappreciated layer of regulation governing neural adaptability during homing behavior. Nevertheless, this hypothesis awaits direct empirical validation. A systematic characterization of isoform-specific TrkB expression and signaling dynamics in the avian brain could thus uncover novel mechanisms underlying cognitive flexibility and adaptive navigation.

### 3.2. Epigenetic Regulation

*BDNF* expression is dynamically regulated by epigenetic mechanisms, primarily DNA methylation and histone post-translational modifications [34]. These covalent modifications alter chromatin conformation at *BDNF* promoter regions without modifying the underlying DNA sequence, thereby modulating transcriptional accessibility. The methylation of CpG dinucleotides—catalyzed by DNA methyltransferases (DNMTs)—is generally associated with transcriptional repression [37]. In contrast, the acetylation of specific lysine residues on histone H3 and H4 tails (e.g., H3K9ac, H3K27ac), mediated by histone acetyltransferases (HATs), correlates with an open chromatin configuration and active gene transcription [38].

In mammalian models, environmental exposures induce a remodeling of the *BDNF* epigenome [39]. Physical exercise and environmental enrichment are linked to reduced CpG methylation and elevated histone acetylation at activity-regulated promoters, particularly those upstream of exons IV and IX, in the hippocampus [40]. These changes coincide with increased *BDNF* mRNA levels and enhanced markers of synaptic plasticity. Conversely, chronic stress or early-life adversity promotes the hypermethylation of *BDNF* promoters and decreased histone acetylation—partly through the upregulation of histone deacetylase (HDAC) expression—resulting in persistent transcriptional downregulation [41,42]. Such epigenetic alterations are associated with deficits in cognitive performance and heightened susceptibility to affective disorders.

In birds, a comprehensive epigenomic annotation of the *BDNF* locus has not been established. Nevertheless, recurrent environmental stimuli—including routine flight training, liberation protocols (“training-and-release”), and competitive racing—constitute relevant experiential inputs that may influence epigenetic states in the avian brain [43]. Comparative studies in other species suggest that structured behavioral regimens can reduce promoter methylation and increase H3/H4 acetylation at neurotrophic gene loci. In pigeons, elevated *BDNF* expression in the HF following navigational training raises the possibility of experience-dependent epigenetic modulation. However, direct evidence linking specific epigenetic marks to *BDNF* transcription in this species remains lacking. Future investigations employing whole-genome bisulfite sequencing (WGBS), reduced representation bisulfite sequencing (RRBS), and chromatin immunoprecipitation followed by sequencing (ChIP-seq) in pigeon neural tissue are necessary to delineate the epigenetic regulatory landscape of *BDNF* and its role in spatial cognition [44].

### 3.3. Post-Transcriptional Regulation by miRNAs

*BDNF* mRNA is subject to post-transcriptional regulation by *microRNAs* (miRNAs), which constitute a rapid and reversible layer of gene expression control [45]. miRNAs bind to complementary sequences within the 3′ untranslated region (3′UTR) of *BDNF* mRNA, leading to transcript degradation or translational repression [46,47]. This mechanism enables a precise modulation of *BDNF* protein levels in response to cellular and environmental cues [46]. Several miRNAs—including *miR-132*, *miR-206*, and *miR-1*—have been experimentally validated as direct regulators of *BDNF* in mammalian systems and are implicated in neurodevelopment, synaptic function, and stress adaptation [47,48,49]. For example, neuronal activity induces *miR-132* expression, which subsequently attenuates *BDNF* translation, forming a negative feedback loop that may contribute to the homeostatic control of neurotrophin signaling [50]. The spatiotemporal specificity of this interaction allows for a localized adjustment of *BDNF* synthesis across distinct brain regions and physiological contexts [46].

The miRNA-mediated regulation of *BDNF* is further embedded within broader non-coding RNA networks. Circular RNAs (circRNAs) and long non-coding RNAs (lncRNAs) can function as competitive endogenous RNAs (ceRNAs) by sequestering miRNAs, thereby indirectly alleviating their repressive effects on target mRNAs [50]. In mammalian models, *circHIPK3* has been shown to bind *miR-124*, preventing its interaction with the *BDNF* 3′UTR and resulting in elevated BDNF protein levels associated with enhanced neuronal survival and axonal growth [51,52]. Conversely, the lncRNA *BDNF-AS* (*BDNF* antisense RNA) suppresses *BDNF* expression through multiple mechanisms, including base-pairing with sense transcripts and the recruitment of Polycomb Repressive Complex 2 (PRC2). PRC2 catalyzes the trimethylation of histone H3 at lysine 27 (H3K27me3), a chromatin modification linked to transcriptional silencing [53].

The pigeon genome contains thousands of annotated non-coding RNA transcripts, but a functional characterization of *BDNF*-associated circRNAs or lncRNAs in this species has not been reported. Given the conservation of core neurotrophic pathways across vertebrates, components of this multi-layered regulatory network may be present in the avian central nervous system. However, direct evidence supporting their role in *BDNF* regulation in pigeons remains absent. Future studies should integrate high-throughput approaches—including whole-transcriptome RNA sequencing (RNA-seq), small RNA-seq, and RNA immunoprecipitation followed by sequencing (RIP-seq or CLIP-seq)—to identify potential *ceRNA* interactions involving BDNF [54,55]. Functional validation using techniques such as in situ hybridization, antisense oligonucleotide knockdown, or CRISPR-based interference (CRISPRi) will be necessary to assess the contribution of non-coding RNA networks to *BDNF* expression dynamics in the brain regions involved in spatial navigation and memory processing [56,57].

## 4. Roles of BDNF in Neural Development in Pigeons

### 4.1. Development of Sensory Systems

In the domestic pigeon, a species with strong visual dependence, BDNF is involved in the development of sensory pathways, particularly the visual system [58]. During ontogeny, *BDNF* and its high-affinity receptor TrkB display region- and stage-specific expression patterns along the retinotectal projection [58]. *BDNF* mRNA is detectable in retinal ganglion cells (RGCs) and dopaminergic amacrine cells. In late embryonic stages, TrkB protein becomes progressively enriched in specific retinal layers, consistent with a role in the maturation of retinal circuitry [59].

The optic tectum functions as the primary visual processing center in birds. By post-hatch day 4 (P4), TrkB expression is predominantly localized in layer 13 of the optic tectum [60]. The in vivo administration of exogenous BDNF via intravitreal injection during this period disrupts normal tectal development, resulting in an abnormal distribution of calbindin-positive neurons and alterations in laminar organization [61]. These neuroanatomical changes are associated with imbalances in interhemispheric visual processing, manifesting as functional asymmetry [62]. Such BDNF-dependent modulation of tectal architecture may contribute to the establishment of lateralized visual functions, which have been observed in pigeons during complex behaviors such as spatial navigation and predator detection.

*BDNF* is also expressed in the avian auditory system [63]. Both *BDNF* and *TrkB* transcripts are detectable in the cochlea during development and adulthood. Following acoustic injury, BDNF may support the survival and metabolic activity of hair cells during regenerative processes. However, exogenous BDNF delivery alone has not been shown to significantly enhance hair cell repair efficiency in experimental models [64]. Additionally, prenatal exposure to structured acoustic stimuli—such as music—has been reported to modulate *BDNF* expression in cortical and sensory-associated regions of hatchlings, with effects persisting into postnatal stages [65]. These findings suggest that early sensory experience can influence neurotrophic signaling, potentially shaping perceptual development.

### 4.2. Development and Regulation of the Motor System

During motor system development in pigeons, BDNF functions as a target-derived neurotrophic factor that influences the survival and maturation of oculomotor neurons [66]. These neurons, which innervate extraocular muscles, express high levels of TrkB receptors during early embryonic stages (e.g., embryonic days 8–12), coinciding with a period of naturally occurring cell death [66]. The in vivo application of exogenous BDNF to the ciliary ganglion or oculomotor nucleus during this window reduces neuronal loss, supporting the formation of oculomotor circuits.

In the context of neuronal maintenance, BDNF acts in concert with other neurotrophic factors, including glial cell line-derived neurotrophic factor (GDNF) and ciliary neurotrophic factor (CNTF) [67,68,69]. Co-administration studies in avian models suggest that these factors may converge on shared intracellular signaling cascades—such as the PI3K/AKT and MAPK pathways—to modulate metabolic activity and axonal transport [70,71]. However, the extent of functional synergy in vivo remains to be fully characterized.

In adult pigeons, BDNF is also implicated in the regulation of neuromuscular junctions between oculomotor nerve terminals and extraocular muscles. Local BDNF signaling has been associated with the organization of acetylcholine receptor clusters and modulation of postsynaptic electrophysiological properties [72,73]. Such trophic influence may contribute to the stability of oculomotor output, which is relevant for reflexive eye movements such as the vestibulo-ocular reflex (VOR). Given the demands of aerial locomotion, sustained neurotrophic support at these synapses could play a role in maintaining motor precision over time [74,75]. Nevertheless, direct evidence linking BDNF-dependent synaptic tuning to flight performance metrics in pigeons has not been established.

## 5. BDNF and Cognition and Behavior

### 5.1. Spatial Navigation and Homing Memory

Long-range homing in pigeons integrates spatial memory, environmental cues, and multisensory inputs [75]. The HF is a central node in this process [75]. *BDNF* is highly expressed in the pigeon HF and has been linked to structural and functional plasticity within this region [2].

The relationship between HF morphology and navigational ability encompasses both innate specializations and potential experience-dependent mechanisms. Comparative neuroanatomical analyses have demonstrated that homing pigeon breeds possess significantly larger HF volumes than non-homing domestic pigeon breeds, a difference attributed to selective breeding for spatial cognition [76]. This constitutes a heritable, breed-specific neural substrate for navigation. Within this specialized lineage, the functional role of BDNF in the HF is critical for navigational performance. A disruption of BDNF/TrkB signaling—via pharmacological blockade—impairs a pigeon’s ability to orient itself using celestial cues (e.g., sun compass) or visual landmarks. This finding underscores the necessity of intact BDNF signaling for processing key navigational information.

The relationship between HF morphology and navigational ability encompasses both innate specializations and potential experience-dependent mechanisms. Comparative neuroanatomical analyses have demonstrated that homing pigeon breeds possess significantly larger HF volumes than non-homing domestic pigeon breeds, a difference attributed to selective breeding for spatial cognition [76]. This constitutes a heritable, breed-specific neural substrate for navigation. It is noteworthy that an analogous link between HF volume and spatial ecology has been robustly established in food-storing birds, such as chickadees and jays, whose reliance on precise cache retrieval has driven the evolution of enlarged hippocampi [77]. While both homing pigeons and food-storing species exemplify adaptive specializations of the avian HF for complex spatial tasks, they represent distinct ecological strategies: the former engages in large-scale, multi-modal navigation over unfamiliar terrain, whereas the latter excels in high-precision, local spatial memory for cached items.

Homing behavior is typically quantified using GPS telemetry [78]. This approach yields ecologically valid metrics, including homing duration, path straightness index (a measure of route directness), and landmark utilization efficiency [78,79]. These parameters provide objective data that can be used to explore potential links between BDNF-related neural processes and navigational performance in naturalistic settings.

### 5.2. BDNF and Stress-Related Behavioral Responses

Pigeons exhibit physiological and behavioral changes in response to environmental challenges such as transport, flight demands, or exposure to potential threats. BDNF has been implicated in the neural adaptations associated with these stressors [80].

An exposure to acute stressors—such as brief handling or sudden auditory stimuli—is associated with transient increases in *BDNF* expression in specific brain regions, which may support short-term adaptive plasticity [81]. In contrast, prolonged or repeated stress exposure (e.g., suboptimal housing conditions or intensive training regimens) correlates with reduced BDNF levels [82]. These reductions are accompanied by structural alterations in stress-responsive circuits and changes in behavior that resemble anxiety-like states in other vertebrates, including decreased exploratory activity and altered social engagement [83].

Conditioned place preference (CPP) assays measure the strength of association between a neutral context and a prior positive or negative experience, providing an index of contextual memory and valence assignment [84]. These metrics offer objective, quantifiable endpoints for evaluating how BDNF-related neural changes correlate with individual differences in stress reactivity.

### 5.3. BDNF and Social Reward-Related Behaviors

Pigeons display social interactions and affiliative behaviors, including pair bonding and coordinated parental care [85]. In other vertebrates, BDNF has been shown to interact with the mesolimbic dopamine system—a network involved in reward processing and social motivation [86,87]. Although direct evidence in pigeons is limited, *BDNF* expression has been detected in striatal and limbic regions homologous to those implicated in social behavior in mammals [88]. During reproductive phases such as courtship or chick rearing, the circulating levels of gonadal steroids and nonapeptides (e.g., mesotocin, an avian homolog of oxytocin) undergo significant changes [89,90]. These hormonal fluctuations coincide with altered *BDNF* expression in brain regions associated with social attachment, suggesting a potential interaction between neuroendocrine and neurotrophic signaling [91]. However, the functional contribution of BDNF to nest-site fidelity or mate preference in pigeons has not been experimentally verified.

Operant conditioning paradigms are commonly used to assess reward-related cognition in pigeons [92,93]. In such assays, birds learn to associate specific visual stimuli (e.g., colors, shapes, or sequential patterns) with food delivery through key-peck responses in controlled chambers. These tasks yield quantifiable measures, including discrimination accuracy, acquisition rate, and reversal learning performance—indices often interpreted as reflecting reinforcement sensitivity and behavioral flexibility. When combined with post-task tissue analysis, these behavioral metrics can be correlated with regional *BDNF* expression to explore its involvement in avian reward processing. Nevertheless, the extent to which BDNF directly modulates dopaminergic signaling or synaptic plasticity in the pigeon striatum requires further investigation.

## 6. Future Perspectives

Future progress in elucidating BDNF’s role in avian cognition will hinge on the development of species-adapted molecular and genetic tools. The absence of targeted neurogenetic approaches—such as the adeno-associated virus-mediated, region-specific manipulation of BDNF or TrkB signaling—currently precludes a causal interrogation of neurotrophic mechanisms in the pigeon brain. Integrating such interventions with single-cell transcriptomics could resolve the identity of BDNF-expressing and BDNF-responsive neuronal populations within navigation-relevant circuits. Concurrently, the potential of peripheral BDNF isoforms (e.g., mBDNF and proBDNF in plasma) as non-invasive biomarkers of central neurotrophic activity merits systematic evaluation, though their physiological relevance to brain function remains unconfirmed. Beyond technical innovation, comparative analyses of *BDNF* gene regulation—spanning promoter architecture, epigenetic landscapes, and non-coding RNA networks—across species with divergent spatial ecologies (e.g., homing pigeons, food-storing birds, and non-spatial specialists) may reveal how neurotrophic systems have been evolutionarily tuned to support distinct cognitive strategies. Such integrative efforts would not only advance our understanding of activity-dependent plasticity in non-mammalian vertebrates but also refine broader models of neural circuit evolution underlying adaptive behavior.

## 7. Conclusions

*BDNF* is expressed in multiple brain regions of pigeons and has been linked to neural development, sensory integration, and spatial behavior. Its coding sequence is highly conserved across vertebrates, while its transcriptional regulation involves multiple promoters and extensive post-transcriptional control by non-coding RNAs—a feature also observed in mammals. Experimental evidence supports a role for BDNF in activity-dependent plasticity within circuits involved in navigation and memory, though most data are derived from correlative or pharmacological studies.

Key gaps persist regarding the causal contribution of BDNF to specific behavioral outputs, the identity of responsive neuronal populations, and the real-time dynamics of its signaling in freely behaving birds. Addressing these questions will require the adaptation of advanced molecular and imaging technologies to avian models. Such efforts may enhance our understanding of neurotrophic function in non-mammalian systems and inform comparative analyses of neural plasticity across vertebrate lineages.

## Data Availability

No new data were created or analyzed in this study. Data sharing is not applicable to this article.

## Abbreviations

The following abbreviations are used in this manuscript:

3′UTR	3′ untranslated region
BDNF	Brain-Derived Neurotrophic Factor
circRNAs	Circular RNAs
CNS	Central nervous system
CREB	cAMP response element-binding protein
ER	Endoplasmic reticulum
HF	Hippocampal formation
LncRNAs	Long non-coding RNAs
LTD	Long-term depression
LTP	Long-term potentiation
MAPK/ERK	Mitogen-activated protein kinase/extracellular signal-regulated kinase
mBDNF	Mature Brain-Derived Neurotrophic Factor
miRNAs	MicroRNAs
MMPs	Matrix metalloproteinases
MSt	Medial striatum
NCL	Nidopallium caudolaterale
NTRK2	Neurotrophic receptor tyrosine kinase 2
p75NTR	p75 neurotrophin receptor
PC1/3	Proprotein convertase 1/3
PI3K/AKT	Phosphatidylinositol 3-kinase/protein kinase B
PLC-γ	Phospholipase C-gamma
proBDNF	Precursor Brain-Derived Neurotrophic Factor
RGCs	Retinal ganglion cells
RNA-seq	RNA sequencing
SNP	Single nucleotide polymorphism
TrkB	Tropomyosin receptor kinase B
TrkB-FL	Full-length tropomyosin receptor kinase B
TrkB-T1	Truncated tropomyosin receptor kinase B isoform 1
TrkB-T2	Truncated tropomyosin receptor kinase B isoform 2
VOR	Vestibulo-ocular reflex

## References

[1] De Luca P., Mele M., Tanqueiro S., Napoli F., Butkevičiūtė U., Souto A.C., Costa R.O., Schwarz A., Drexel M., Sebastião A.M. (2025). Synaptic Accumulation of GluN2B-Containing NMDA Receptors Mediates the Effects of BDNF-TrkB Signalling on Synaptic Plasticity and in Hyperexcitability during Status Epilepticus. J. Biomed. Sci..

[2] Kowiański P., Lietzau G., Czuba E., Waśkow M., Steliga A., Moryś J. (2018). BDNF: A key factor with multipotent impact on brain signaling and synaptic plasticity. Cell. Mol. Neurobiol..

[3] Sizemore B.A., Bausher A., Paul E., Russell M., Bingman V.P. (2022). Space, feature, and risk sensitivity in homing pigeons (*Columba livia*): Broadening the conversation on the role of the avian hippocampus in memory. Learn. Behav..

[4] Güntürkün O., von Eugen K., Packheiser J., Pusch R. (2021). Avian pallial circuits and cognition: A comparison to mammals. Curr. Opin. Neurobiol..

[5] Herold C., Coppola V.J., Bingman V.P. (2015). The maturation of research into the avian hippocampal formation: Recent discoveries from one of the nature’s foremost navigators. Hippocampus.

[6] Aid T., Kazantseva A., Piirsoo M., Palm K., Timmusk T. (2007). Mouse and rat *BDNF* gene structure and expression revisited. J. Neurosci. Res..

[7] Esvald E.-E., Avarlaid A., Koppel I., Tuvikene J., Timmusk T. (2026). Making of BDNF: Role of promoters, enhancers, and untranslated regions. Trends Neurosci..

[8] You H., Lu B. (2023). Diverse functions of multiple Bdnf transcripts driven by distinct Bdnf promoters. Biomolecules.

[9] Keifer J. (2021). Comparative Genomics of the *BDNF* Gene, Non-Canonical Modes of Transcriptional Regulation, and Neurological Disease. Mol. Neurobiol..

[10] Pruunsild P., Kazantseva A., Aid T., Palm K., Timmusk T. (2007). Dissecting the human BDNF locus: Bidirectional transcription, complex splicing, and multiple promoters. Genomics.

[11] National Center for Biotechnology Information *Columba livia* Isolate bColLiv1 Breed Racing Homer Chromosome 5, bColLiv1.pat.W.v2, Whole Genome Shotgun Sequence. Nucleotide–NCBI. https://www.ncbi.nlm.nih.gov/nuccore/NC_088606.1.

[12] Zhang G., Li C., Li Q., Li B., Larkin D.M., Lee C., Storz J.F., Antunes A., Greenwold M.J., Meredith R.W. (2014). Comparative Genomics Reveals Insights into Avian Genome Evolution and Adaptation. Science.

[13] Pruunsild P., Sepp M., Orav E., Koppel I., Timmusk T. (2011). Identification of cis-elements and transcription factors regulating neuronal activity-dependent transcription of human *BDNF* gene. J. Neurosci..

[14] Qiu Y., Zhu L., Cai W., Zhu L. (2025). Research Progress on BDNF and Depression. ACS Chem. Neurosci..

[15] Das S., Vera M., Gandin V., Singer R.H., Tutucci E. (2021). Intracellular mRNA transport and localized translation. Nat. Rev. Mol. Cell Biol..

[16] Thomas A.X., Del Angel Y.C., Gonzalez M.I., Carrel A.J., Carlsen J., Lam P.M., Hempstead B.L., Russek S.J., Brooks-Kayal A.R. (2016). Rapid Increases in proBDNF After Pilocarpine-Induced Status Epilepticus in Mice Are Associated with Reduced proBDNF Cleavage Machinery. eNeuro.

[17] Sen A., Nelson T.J., Alkon D.L. (2017). ApoE Isoforms Differentially Regulate Cleavage and Secretion of BDNF. Mol. Brain.

[18] Yamada M., Hayashi H., Yuuki M., Matsushima N., Yuan B., Takagi N. (2018). Furin Inhibitor Protects Against Neuronal Cell Death Induced by Activated NMDA Receptors. Sci. Rep..

[19] Foltran R.B., Diaz S.L. (2016). BDNF isoforms: A round trip ticket between neurogenesis and serotonin?. J. Neurochem..

[20] Eggert S., Kins S., Endres K., Brigadski T. (2021). Brothers in Arms: proBDNF/BDNF and sAPPα/Aβ-Signaling and Their Common Interplay with ADAM10, TrkB, p75NTR, Sortilin, and SorLA in the Progression of Alzheimer’s disease. Biol. Chem..

[21] Zhang Y., Bai X., Zhang Y., Yao S., Cui Y., You L.H., Yu P., Chang Y.C., Gao G. (2022). Hippocampal iron accumulation impairs synapses and memory via suppressing furin expression and downregulating BDNF maturation. Mol. Neurodegener..

[22] Legutko D., Bijoch Ł., Olszak G., Kuźniewska B., Kalita K., Yasuda R., Kaczmarek L., Michałuk P. (2025). BDNF-driven synaptic plasticity requires autocrine matrix metalloproteinase-9 activity. Sci. Adv..

[23] Cao W., Duan J., Wang X., Zhong X., Hu Z., Huang F., Wang H., Zhang J., Li F., Zhang J. (2014). Early enriched environment induces an increased conversion of proBDNF to BDNF in the adult rat’s hippocampus. Behav. Brain Res..

[24] Faria R.S., Sartori C.R., Canova F., Ferrari E.A.M. (2013). Classical aversive conditioning induces increased expression of mature-BDNF in the hippocampus and amygdala of pigeons. Neuroscience.

[25] Brenowitz E.A. (2013). Testosterone and brain-derived neurotrophic factor interactions in the avian song control system. Neuroscience.

[26] Suzuki K., Maekawa F., Suzuki S., Nakamori T., Sugiyama H., Kanamatsu T., Tanaka K., Ohki-Hamazaki H. (2012). Elevated expression of brain-derived neurotrophic factor facilitates visual imprinting in chicks. J. Neurochem..

[27] Jiang S., Li H., Zhang L., Mu W., Zhang Y., Chen T., Wu J., Tang H., Zheng S., Liu Y. (2025). Generic Diagramming Platform (GDP): A comprehensive database of high-quality biomedical graphics. Nucleic Acids Res..

[28] Wang C.S., Kavalali E.T., Monteggia L.M. (2022). BDNF signaling in context: From synaptic regulation to psychiatric disorders. Cell.

[29] Hashimoto K. (2015). Brain-derived neurotrophic factor (BDNF) and its precursor proBDNF as diagnostic biomarkers for major depressive disorder and bipolar disorder. Eur. Arch. Psychiatry Clin. Neurosci..

[30] Moya-Alvarado G., Valero-Peña X., Aguirre-Soto A., Bustos F.J., Lazo O.M., Bronfman F.C. (2024). PLC-γ-Ca^2+^ pathway regulates axonal TrkB endocytosis and is required for long-distance propagation of BDNF signaling. Front. Mol. Neurosci..

[31] Reichardt L.F. (2006). Neurotrophin-regulated signalling pathways. Philos. Trans. R. Soc. Lond B Biol. Sci..

[32] Mitrovic M., Selakovic D., Jovicic N., Ljujic B., Rosic G. (2025). BDNF/proBDNF interplay in the mediation of neuronal apoptotic mechanisms in neurodegenerative diseases. Int. J. Mol. Sci..

[33] Duarte-Ruiz M., Morelo-Castillo A., El Yousfi Y., Moreno-Castro C., Martínez-Martínez N., Jiménez-Lozano S., Kennel M., Ruiz-Rodríguez C., Rodríguez-López A., López-Ros J. (2025). Regulation of NTRK2 alternative splicing by PRPF40B controls neural differentiation and synaptic plasticity. Cell Death Dis..

[34] Baxter G.T., Radeke M.J., Kuo R.C., Makrides V., Hinkle B., Hoang R., Medina-Selby A., Coit D., Valenzuela P., Feinstein S.C. (1997). Signal transduction mediated by the truncated trkB receptor isoforms, trkB.T1 and trkB.T2. J. Neurosci..

[35] Cao T., Matyas J.J., Renn C.L., Faden A.I., Dorsey S.G., Wu J. (2020). Function and mechanisms of truncated BDNF receptor TrkB.T1 in neuropathic pain. Cells.

[36] Chen K.W., Chen L. (2017). Epigenetic regulation of *BDNF* gene during development and diseases. Int. J. Mol. Sci..

[37] Lyko F. (2018). The DNA methyltransferase family: A versatile toolkit for epigenetic regulation. Nat. Rev. Genet..

[38] Devaiah B.N., Case-Borden C., Gegonne A., Hsu C.H., Chen Q., Meerzaman D., Dey A., Ozato K., Singer D.S. (2016). BRD4 is a histone acetyltransferase that evicts nucleosomes from chromatin. Nat. Struct. Mol. Biol..

[39] Costa G.A., Silva N.K.G.T., Marianno P., Chivers P., Bailey A., Camarini R. (2023). Environmental enrichment increased Bdnf transcripts in the prefrontal cortex: Implications for an epigenetically controlled mechanism. Neuroscience.

[40] Borba L.A., Broseghini L.D.R., Manosso L.M., de Moura A.B., Botelho M.E.M., Arent C.O., Behenck J.P., Hilsendeger A., Kammer L.H., Valvassori S.S. (2021). Environmental enrichment improves lifelong persistent behavioral and epigenetic changes induced by early-life stress. J. Psychiatr. Res..

[41] Kundakovic M., Gudsnuk K., Herbstman J.B., Tang D., Perera F.P., Champagne F.A. (2015). DNA methylation of BDNF as a biomarker of early-life adversity. Proc. Natl. Acad. Sci. USA.

[42] Fuchikami M., Morinobu S., Kurata A., Yamamoto S., Yamawaki S. (2009). Single immobilization stress differentially alters the expression profile of transcripts of the brain-derived neurotrophic factor (BDNF) gene and histone acetylation at its promoters in the rat hippocampus. Int. J. Neuropsychopharmacol..

[43] Guyonnet A.E.M., Racicot K.J., Brinkman B., Iwaniuk A.N. (2024). The quantitative anatomy of the hippocampal formation in homing pigeons and other pigeon breeds: Implications for spatial cognition. Brain Struct. Funct..

[44] Firdaus Z., Li X. (2024). Epigenetic explorations of neurological disorders, the identification methods, and therapeutic avenues. Int. J. Mol. Sci..

[45] Bhat V.D., Jayaraj J., Babu K. (2022). RNA and neuronal function: The importance of post-transcriptional regulation. Oxf. Open Neurosci..

[46] Vicario A., Colliva A., Ratti A., Davidovic L., Baj G., Gricman L., Colombrita C., Pallavicini A., Jones K.R., Bardoni B. (2015). Dendritic targeting of short and long 3′ UTR BDNF mRNA is regulated by BDNF or NT-3 and distinct sets of RNA-binding proteins. Front. Mol. Neurosci..

[47] Miao Z., Mao F., Liang J., Szyf M., Wang Y., Sun Z.S. (2018). Anxiety-related behaviours associated with microRNA-206-3p and *BDNF* expression in pregnant female mice following psychological social stress. Mol. Neurobiol..

[48] Li E.Y., Zhao P.J., Jian J., Yin B.Q., Sun C.Y., Xu C.X., Tang Y.C., Wu H. (2019). Vitamin B1 and B12 mitigates neuron apoptosis in cerebral palsy by augmenting *BDNF* expression through MALAT1/miR-1 axis. Cell Cycle.

[49] Aten S., Hansen K.F., Hoyt K.R., Obrietan K. (2016). The miR-132/212 locus: A complex regulator of neuronal plasticity, gene expression and cognition. RNA Dis..

[50] Vo N., Klein M.E., Varlamova O. (2005). A cAMP-response element binding protein-induced microRNA regulates neuronal morphogenesis. Proc. Natl. Acad. Sci. USA.

[51] Chen M., Lai X., Wang X., Ying J., Zhang L.L., Zhou B., Liu X., Zhang J., Wei G., Hua F.Z. (2021). Long non-coding RNAs and circular RNAs: Insights into microglia and astrocyte mediated neurological diseases. Front. Mol. Neurosci..

[52] De Assis G.G., Murawska-Ciałowicz E. (2024). BDNF Modulation by microRNAs: An Update on the Experimental Evidence. Cells.

[53] Wang H., Xiong X., Wang J., Wang Z., Li Y. (2023). CircHIPK3 promotes neuroinflammation through regulation of the miR-124-3p/STAT3/NLRP3 signaling pathway in Parkinson’s disease. Adv. Clin. Exp. Med..

[54] Bohnsack J.P., Teppen T., Kyzar E.J., Dzitoyeva S., Pandey S.C. (2019). The lncRNA BDNF-AS is an epigenetic regulator in the human amygdala in early onset alcohol use disorders. Transl. Psychiatry.

[55] Bottini S., Pratella D., Grandjean V., Repetto E., Trabucchi M. (2018). Recent computational developments on CLIP-seq data analysis and microRNA targeting implications. Brief. Bioinform..

[56] Tay Y., Rinn J., Pandolfi P.P. (2014). The multilayered complexity of ceRNA crosstalk and competition. Nature.

[57] Colliva A., Maynard K.R., Martinowich K., Tongiorgi E. (2019). Detecting single and multiple BDNF transcripts by in situ hybridization in neuronal cultures and brain sections. Brain-Deriv. Neurotrophic Factor (BDNF).

[58] Mysona B.A., Zhao J., Bollinger K.E. (2017). Role of BDNF/TrkB pathway in the visual system: Therapeutic implications for glaucoma. Expert Rev. Ophthalmol..

[59] Liu X., Grishanin R.N., Tolwani R.J., Rentería R.C., Xu B., Reichardt L.F., Copenhagen D.R. (2007). Brain-derived neurotrophic factor and TrkB modulate visual experience-dependent refinement of neuronal pathways in retina. J. Neurosci..

[60] Fernández M., Morales C., Durán E., Fernández-Colleman S., Sentis E., Mpodozis J., Karten H.J., Marín G.J. (2020). Parallel organization of the avian sensorimotor arcopallium: Tectofugal visual pathway in the pigeon (*Columba livia*). J. Comp. Neurol..

[61] Cohen-Cory S., Kidane A.H., Shirkey N.J., Marshak S. (2010). Brain-derived neurotrophic factor and the development of structural neuronal connectivity. Dev. Neurobiol..

[62] Manns M., Freund N., Leske O., Güntürkün O. (2008). Breaking the balance: Ocular BDNF-injections induce visual asymmetry in pigeons. Dev. Neurobiol..

[63] McLellan K., Sabbagh S., Takahashi M., Hong H., Wang Y., Sanchez J.T. (2024). BDNF differentially affects low- and high-frequency neurons in a primary nucleus of the chicken auditory brainstem. Biology.

[64] Tisi A., Rovers J., Vink H.A., Ramekers D., Maccarone R., Versnel H. (2021). No protective effects of hair cells or supporting cells in ototoxically deafened guinea pigs upon administration of BDNF. Brain Sci..

[65] Kathpalia P., Nag T.C., Chattopadhyay P., Sharma A., Bhat M.A., Roy T.S., Wadhwa S. (2019). In ovo sound stimulation mediated regulation of BDNF in the auditory cortex and hippocampus of neonatal chicks. Neuroscience.

[66] Steljes T.P., Kinoshita Y., Wheeler E.F., Oppenheim R.W., von Bartheld C.S. (1999). Neurotrophic factor regulation of developing avian oculomotor neurons: Differential effects of BDNF and GDNF. J. Neurobiol..

[67] Mashanov V., Alwan A., Kim M.W., Lai D., Poerio A., Ju Y.M., Kim J.H., Yoo J.J. (2020). Synergistic effect of CNTF and GDNF on directed neurite growth in chick embryo dorsal root ganglia. PLoS ONE.

[68] Hu Y., Grodzki L.M., Bartsch U. (2025). Survival and axonal regeneration of retinal ganglion cells in a mouse optic nerve crush model after a cell-based intravitreal co-administration of ciliary neurotrophic factor and glial cell line-derived neurotrophic factor at different post-lesion time points. Cells.

[69] Dulz S., Bassal M., Flachsbarth K., Riecken K., Fehse B., Schlichting S., Bartsch S., Bartsch U. (2020). Intravitreal co-administration of GDNF and CNTF confers synergistic and long-lasting protection against injury-induced cell death of retinal ganglion cells in mice. Cells.

[70] Olivares-Hernández J.D., Carranza M., Balderas-Márquez J.E., Epardo D., Baltazar-Lara R., Ávila-Mendoza J., Martínez-Moreno C.G., Luna M., Arámburo C. (2022). Neuroprotective and regenerative effects of growth hormone (GH) in the embryonic chicken cerebral pallium exposed to hypoxic-ischemic (HI) injury. Int. J. Mol. Sci..

[71] Mantilla C.B., Ermilov L.G., Greising S.M., Gransee H.M., Zhan W.Z., Sieck G.C. (2023). Electrophysiological effects of BDNF and TrkB signaling at type-identified diaphragm neuromuscular junctions. J. Neurophysiol..

[72] Zhang J., Kwan H.L.R., Chan C.B., Lee C.W. (2025). Localized release of muscle-generated BDNF regulates the initial formation of postsynaptic apparatus at neuromuscular synapses. Cell Death Differ..

[73] Just-Borràs L., Cilleros-Mañé V., Hurtado E., Biondi O., Charbonnier F., Tomàs M., Garcia N., Tomàs J., Lanuza M.A. (2021). Running and swimming differently adapt the BDNF/TrkB pathway to a slow molecular pattern at the NMJ. Int. J. Mol. Sci..

[74] Davis-López de Carrizosa M.A., Morado-Díaz C.J., Tena J.J., Benítez-Temiño B., Pecero M.L., Morcuende S.R., de la Cruz R.R., Pastor A.M. (2009). Complementary actions of BDNF and neurotrophin-3 on the firing patterns and synaptic composition of motoneurons. J. Neurosci..

[75] Gagliardo A., Bingman V.P. (2024). The avian olfactory system and hippocampus: Complementary roles in the olfactory and visual guidance of homing pigeon navigation. Curr. Opin. Neurobiol..

[76] Mehlhorn J., Rehkämper G. (2009). Neurobiology of the homing pigeon—A review. Naturwissenschaften.

[77] Bingman V.P., Siegel J.J., Gagliardo A., Erichsen J.T. (2006). Representing the Richness of Avian Spatial Cognition: Properties of a Lateralized Homing Pigeon Hippocampus. Rev. Neurosci..

[78] Cioccarelli S., Giunchi D., Pollonara E., Casini G., Bingman V.P., Gagliardo A. (2024). GPS tracking technology and re-visiting the relationship between the avian visual Wulst and homing pigeon navigation. Behav. Brain Res..

[79] Taylor L.A., Portugal S.J., Biro D. (2017). Homing pigeons (*Columba livia*) modulate wingbeat characteristics as a function of route familiarity. J. Exp. Biol..

[80] Li S., Tang Z., Li M., Yang L., Shang Z. (2025). Neural Correlates of Flight Acceleration in Pigeons: Gamma-Band Activity and Local Functional Network Dynamics in the AId Region. Animals.

[81] Brivio P., Sbrini G., Riva M.A., Calabrese F. (2020). Acute Stress Induces Cognitive Improvement in the Novel Object Recognition Task by Transiently Modulating Bdnf in the Prefrontal Cortex of Male Rats. Cell. Mol. Neurobiol..

[82] Ping G., Qian W., Song G., Zhaochun S. (2014). Valsartan reverses depressive/anxiety-like behavior and induces hippocampal neurogenesis and expression of BDNF protein in unpredictable chronic mild stress mice. Pharmacol. Biochem. Behav..

[83] Hegde A., Suresh S., Mitra R. (2020). Early-life short-term environmental enrichment counteracts the effects of stress on anxiety-like behavior, brain-derived neurotrophic factor and nuclear translocation of glucocorticoid receptor in the basolateral amygdala. Sci. Rep..

[84] McKendrick G., Graziane N.M. (2020). Drug-Induced Conditioned Place Preference and Its Practical Use in Substance Use Disorder Research. Front. Behav. Neurosci..

[85] Jankowiak Ł., Tryjanowski P., Hetmański T., Skórka P. (2018). Experimentally evoked same-sex sexual behaviour in pigeons: Better to be in a female-female pair than alone. Sci. Rep..

[86] Koo J.W., Labonté B., Engmann O., Calipari E.S., Juarez B., Lorsch Z., Walsh J.J., Friedman A.K., Yorgason J.T., Han M.H. (2016). Essential role of mesolimbic brain-derived neurotrophic factor in chronic social stress-induced depressive behaviors. Biol. Psychiatry.

[87] Zhang H., Qian Y.L., Li C., Liu D., Wang L., Wang X.Y., Liu M.J., Liu H., Zhang S., Guo X.Y. (2017). Brain-derived neurotrophic factor in the mesolimbic reward circuitry mediates nociception in chronic neuropathic pain. Biol. Psychiatry.

[88] Jarvis E.D., Güntürkün O., Bruce L., Csillag A., Karten H., Kuenzel W., Medina L., Paxinos G., Perkel D.J., Shimizu T. (2005). Avian brains and a new understanding of vertebrate brain evolution. Nat. Rev. Neurosci..

[89] Jurek B., Neumann I.D. (2018). The oxytocin receptor: From intracellular signaling to behavior. Physiol. Rev..

[90] Tobari Y., Tsutsui K. (2019). Effects of social information on the release and expression of gonadotropin-inhibitory hormone in birds. Front. Endocrinol..

[91] Maynard K.R., Hobbs J.W., Phan B.D.N., Gupta A., Rajpurohit S., Williams C., Rajpurohit A., Shin J.H., Jaffe A.E., Martinowich K. (2018). BDNF-TrkB signaling in oxytocin neurons contributes to maternal behavior. eLife.

[92] McMillan N., Roberts W.A. (2015). A three-stimulus midsession reversal task in pigeons with visual and spatial discriminative stimuli. Anim. Cogn..

[93] Delius J.D., Delius J.A.M. (2019). Systematic analysis of pigeons’ discrimination of pixelated stimuli: A hierarchical pattern recognition system is not identifiable. Sci. Rep..

